# A Peculiar Case of Phthisis

**Published:** 1872-03-01

**Authors:** Norman Bridge

**Affiliations:** 229 W. Madison St.; Chicago


### A PECULIAR CASE OF PHTHISIS.

BY NORMAN BRIDGE, M. D., CHICAGO.

The case detailed below is reported as well for its lesson in diagnosis, as for the fact of its differing from ordinary cases of consumption.

Mrs. V., past middle age, a slim, nervous woman, had early in life enjoyed excellent health; a dozen years ago she had uterine disease which had subsided on the cessation of menstruation. She had, however, complained of dyspepsia for a long time, which had, during the two last years, steadily grown worse. She had endeavored to cure herself by dieting, cold water treatment, and eating of coarse bread—really a system of starvation—having, under it, grown worse instead of better; she had wasted to little more than a skeleton, and was unable to sit up when the writer was asked to see her. She complained of absolutely no symptoms not directly referable to the stomach and bowels. She had indigestion, feeling of weight and fulness in the stomach after eating, sensations of fever, often great drowsiness, and occasionally vomiting. The appetite—the disposition to eat—was good, and she continually reproached herself for eating too much—a fault she supposed herself guilty of—carefully eschewing all animal food on the ground that it was "too heavy." There was, most of the time, constipation, and the patient was inclined to despondency of mind. She had some pain, at times, in the epigastrium.

A cursory examination of the lungs was made, more because it seemed natural in an examination of a patient not to pass them by, than because any symptoms had referred to them; but the upper half of the chest only was examined.

The respiration was loud and puerile on both sides, with no bronchial breathing, no prolongation of expiratory sound. There was no rhoncus or rattling, no fluid sound; there was no whistling or any sound abnormal, save the loud respiratory murmur; the inspiration was regular and not jerking. The resonance, on percussion, was alike on the two sides, and was thought to be normal. All the sounds, by auscultation and percussion, were absolutely alike on both sides of that part of the chest examined—not the slightest difference could be detected between them.

There had been no hæmoptysis, cough or expectoration whatever—no symptoms to direct attention to the lungs, so they were not examined further. The puerile sound, in the absence of bronchial breathing and rhoncus, was supposed to be made apparent by the absence of soft tissue over the bony chest wall—hardly anything but skin covering the ribs.

Catarrhal irritation of the stomach was diagnosed, and the patient was advised to take animal foods, in liquid form, abundantly; a course of tonic treatment was also urged.

In about a month word was sent me that the patient was dead, and that a *post mortem* examination would be allowed.

On arriving at the home of the subject of the examination I learned that from the time I saw her until her death there was no material change in her symptoms except in the occurrence of one paroxysm of coughing several days before her death, and diarrhœa during two days previous to death. There had been no hectic, or night sweats; no exacerbations of fever.

At the autopsy the stomach was found somewhat dilated near its pyloric orifice—the pylorus being thickened, and the opening contracted. The gastric mucous membrane was slightly thickened, and at the dilated portion gave evidence of a slight degree of chronic inflammation. The small intestines were, in several places, contracted, as though by former chronic inflammation of the intestinal walls, and the part of the intestine superior to each constriction was dilated to twice its normal calibre. There were more extensive contractions in the large intestines—the whole of the descending colon being condensed to the size of an adult's little finger, with the broad dilation above the constriction. There was no evidence of recent inflammation in the mucous membrane of any of the intestines.

There were signs of former inflammation in a part of the right lobe of the liver, but there was no fatty degeneration.

The lungs were filled with tubercles, most of them grey tubercles, and a portion with yellow centres; the masses were small.

The deposit filled all parts of both lungs; there was no spot uninvaded, and there could not have been more than one-half the true lung substance that was in fit condition for respiratory function at the time of death.

In the lower part of both lungs were a few small cavities filled with purulent matter; none were found above the lower third, and aside from these the deposit was about equally distributed to all portions of the lungs.

The pleuræ were attached in many places by adhesions that were evidently old.

229 W. Madison St.

